# Comparison of double filtration plasmapheresis with immunoadsorption therapy in patients with anti-glomerular basement membrane nephritis

**DOI:** 10.1186/1471-2369-15-128

**Published:** 2014-08-03

**Authors:** Yi-yan Zhang, Zheng Tang, Dong-mei Chen, De-hua Gong, Da-xi Ji, Zhi-hong Liu

**Affiliations:** 1 National Clinical Research Center of Kidney Diseases, Jinling Hospital, Nanjing University School of Medicine, Nanjing 210002, P. R. China

**Keywords:** Double filtration plasmapheresis (DFPP), Immunoadsorption (IA), Anti-GBM disease

## Abstract

**Background:**

Double filtration plasmapheresis (DFPP) and (IA) are both used to clear antibody. However, the clinical efficacy and safety of DFPP in patients with anti-glomerular basement membrane (anti-GBM) disease are unclear.

**Methods:**

The 28 enrolled patients diagnosed serologically and pathologically with anti-GBM disease from 2003 to 2013 included 16 treated with DFPP and 12 with IA, with all patients administered immunosuppressive agents. DFPP consisted of an EC50W filter for plasma separation and an EC20W filter for plasma fractionation. A double volume of plasma was processed, and each patient received a 30–40 g human albumin supplement during each session. IA consisted of 10 cycles per session, with 8–10 sessions performed daily or every other day and each session regenerating 30–60 L of plasma. Serum anti-GBM antibodies and IgG were measured, and urinary and blood tests were performed, before and after each procedure. Renal function and outcome were determined.

**Results:**

The 28 patients consisted of 13 males and 15 females, of median age 44.5 years (range, 22.5–57 years). Six patients had pulmonary hemorrhage and 18 had serum creatinine concentrations >500 umol/L. The average serum creatinine concentration at early onset of disease was 525 umol/L while the peak concentration was 813 umol/L. All patients showed progressive increases in serum creatinine and required CRRT during the course of disease. Pathological examination showed an average 73.9% of crescents (range, 54.6–95.4%).The clinical and pathological features of the DPPP and IA groups were similar. Efficacy of clearing anti-GBM antibody was similar in the two groups (59.0 vs. 71.2%, P = 1.00), although fewer patients in the DFPP group experienced reduced IgG (62.7 vs. 83.5%, p = 0.002). One patient each had a pulmonary hemorrhage and a subcutaneous hemorrhage during treatment, but there were no other serious complications. At the end of follow-up, patient survival and renal survival were similar in the DFPP and IA groups.

**Conclusion:**

DPPP plus immunosuppressive therapy efficiently and safely removed anti-GBM antibodies. The fewer plasma-associated side effects and reduced loss of IgG suggest that DFPP may be a better treatment choice for anti-GBM disease, especially in patients with insufficient plasma.

## Background

Anti-GBM nephritis is an autoimmune disorder characterized by rapidly progressive glomerulonephritis and the presence of circulating anti-glomerular basement membrane (anti-GBM) antibodies. If accompanied by pulmonary hemorrhage, this disease is often called Goodpasture’s syndrome, with a high mortality rate [1,2]. Treatment consists of immunosuppressive agents and removal of circulating antibodies. Extracorporeal removal of anti-GBM antibody, by, for example, plasmapheresis or immunoadsorption (IA) is an effective treatment for Goodpasture’s syndrome. However, lack ofimmunoadsorption column limits the clinical applications of this method. In contrast, double filtration plasmapheresis (DFPP) requires smaller amounts of plasma or albumin and has been used to remove auto-antibodies, particularly those associated with immunologic pathogenesis, included diseases such as myasthenia gravis, chronic inflammatory neuropathy, Guillain-Barré syndrome, and ANCA associated vasculitides. Recently, DFPP was used to in several patients with anti-GBM nephritis to remove serum anti-GBM antibodies. To expand on these findings, we compared the effects of DFPP and immunoadsorption (IA) on serum anti-GBM concentrations, as well as comparing their clinical efficacy in patients with anti-GBM nephritis.

## Methods

### Patients

Patients diagnosed with anti-GBM nephritis and hospitalized between December 2003 and June 2013 in the Department of Nephrology, Jinling Hospital were considered eligible. Other inclusion criteria included a rapid decrease in renal function, with persistent hematuria and anuria, and an ultrasound showing normal kidney size; positivity for serum anti-GBM antibody; the renal biopsy showing crescentic glomerulonephritis with IF liner IgG deposition along the glomerular basement membrane (GBM). Clinical and pathologic data were collected from medical records at the time of presentation and during follow-up. Of 28 patients who underwent plasma therapy, 16 underwent DFPP and 12 underwent staphylococcal protein A immunoadsorption. Patients with active infection, immunodeficiency, or severe cardiovascular or cerebrovascular disease were excluded. Patients underwent DFPP, staphylococcal protein A immunoadsorption and renal biopsy for clinical not research purposes. All patients or their parent/guardian provided written informed consent. The study protocol was approved by Medical Ethics Committee of Jinling Hospital.

### Treatment and fellow-up protocols

All 28 patients received methylprednisolone pulse therapy, followed by oral prednisone or mycophenolate mofetil (MMF) or intravenous cyclophosphamide pulse therapy. Patients showing deterioration of renal function received maintenance hemodialysis therapy. Each patient in the DFPP group underwent 2–4 sessions every other day. IA was administered for 10 cycles per session, with 8–10 sessions performed daily or once every other day; a total of 30–60 L of plasma was regenerated during each session. Serum anti-GBM antibodies and serum IgG were measured after each procedure.

Follow-up data were obtained from patients’ medical records. The primary outcomes were patient and renal survival.

### Methods of DFPP and IA

1.5 or 2 plasma volumes was processed during each DFPP session. Two filters were used, an EC50W filter (Asahi Kasei Corporation, Japan) as the first filter for plasma separation and the second and EC20W filter (Asahi Kasei) for plasma fractionation. Using a blood pump, native blood was pumped into the first filter and separated into plasma and cellular components. The second pump was used to pump filtered plasma into the second filter, which separated albumin from larger plasma molecules, including immunoglobulins, immune complexes, and lipoproteins. Low molecular weight heparin was used for anticoagulants. The blood flow was set to 100 ~ 120 ml/min. During each session, each patient received a supplement of 30–40 g human albumin. During DFPP, waste plasma was discarded intermittently. When the pressure on the second filter reached the threshold value to discard plasma, 800 ml normal saline were used to flush the second filter of accumulated plasma proteins, prior to discarding waste plasma. After each session, 200 ~ 400 ml fresh frozen plasma was used for supplement.

IA was performed using Immunosorba PH-350 (Asahi Kasei Medical, Tokyo, Japan). Each course of treatment consisted of three sessions of IA. Each session was carried out daily or every other day. Three litres of plasma was processed at each session. Anticoagulant was the same as DFPP. No plasma substitution was needed as the clean plasma was returned to the patient.

### Histopathology

For light microscopy, renal biopsy specimens were fixed in 10% neutral buffered formalin, embedded in paraffin and sectioned at 2 μm thickness. Sections were stained with hematoxylin-eosin, periodic acid-Schiff’s reagent, Masson’s trichrome and periodic acid methenamine silver. Glomerular, tubular interstitial and vascular lesions in biopsies were recorded. Biopsy samples were evaluated separately by two pathologists, with each blinded to the other’s results and to patients’ data.

### Statistical methods

All data were analyzed using SPSS program 16.0 for Windows. Results were expressed as means ± standard deviation or median, depending on whether the data were normally distributed. Between group differences were evaluated statistically using t-tests, whereas rates were compared by using the Kruskal-Wallis H test or the χ2 test. A p-value <0.05 indicated statistical significance.

## Results

### Clinical and pathologic data

The 28 patients included 13 males and 15 females, of median age 44.5 years (range, 9–68 years). The median duration of disease was 5 weeks (range, 1 week–5 years). Six patients had pulmonary hemorrhage, including one diagnosed with diffuse alveolar hemorrhage and five with blood-tinged sputum. Three patients were ANCA positive, 10 had anuria and 18 had a serum creatinine (SCr) concentration > 500 μmol/l and required renal replacement therapy at disease onset. Thirteen patients had hypertension, and 17 had symptoms of gross hematuria. There were no significant differences between the DFPP and IA groups (Table 1).

**Table 1:** Baseline demographic and clinical characteristics of the DFPP and IA groups

	**DFPP group (n = 16)**	**IA group(n = 12)**	**P**
Sex (Male/Female)	7/9	6/6	1.000
Age, yr	40 (21.8 ~ 50.2)	49.0 (27.5 ~ 60.3)	0.150
Duration of disease (wks)	5 (3 ~ 9)	8 (3 ~ 10)	0.056
Duration of kidney disease (wks)	2 (1.5 ~ 9)	2 (2 ~ 3)	0.241
Exposure to chemicals (%)	3 (18.8%)	0	0.238
Pulmonary hemorrhage (%)	4 (25.0%)	2 (16.7%)	0.657
Smoking (%)	4 (25.0%)	4 (33.3%)	0.691
Gross hematuria (%)	10 (62.5%)	5 (41.7%)	0.445
Anuria (%)	8 (50.0%)	2 (16.7%)	0.114
Anemia (%)	15 (93.8%)	11 (91.7%)	1.000
RRT at onset (%)	5 (31.2%)	2 (16.7%)	0.662
Hypertension (%)	7 (43.8%)	6 (50.0%)	1.000

RRT, renal replacement therapy.

Laboratory and pathological data are shown in Table 2. Most patients were anemic upon admission to hospital, 19 (67.9%) had disorders of calcium and phosphorus metabolism, 18 (64.3%) had high C-reactive protein (CRP) concentrations. Except for patients with anuria, all had abnormal findings on urinalysis, including 7 (25.0%) with a nephrotic degree of proteinuria. Upon hospital admission, 18 (64.3%) patients had SCr levels >500 μmol/L, with 26 (92.9%) having SCr levels >500 μmol/L during the course of disease. NAG and RBP were also higher than normal. Except for NAG, which was significantly higher in the DPP than in the IA group (84.4 ± 37.3 vs. 58.1 ± 33.7, p = 0.03), none of these data differed significantly in the two groups. At the time of biopsy, a median 73.9% (range, 54.6–95.4%) of glomeruli per patient had a crescent shape, with 26 (92.9%) patients having >50% and 11 (39.3%) having >85% crescent-shaped glomeruli.

**Table 2:** Laboratory and pathological findings in the DFPP and IA groups

	**DFPP group (n = 15)**	**IA group (n = 12)**	**p**
WBC (10^9^/L)	9.39 ± 4.64	8.25 ± 3.17	0.458
Hemoglobin (g/dl)	85.4 ± 19.6	85.1 ± 13.0	0.626
Platelets (10^9^/L)	297 ± 174	235 ± 127	0.137
Urin protein (g/24 hr)	2.85 ± 1.74	1.59 ± 1.36	0.053
Microscopic hematuria (10^5^/ml)	2200 (950 ~ 5000)	1725 (1300 ~ 2965)	0.207
Serum albumin (g/L)	34.6 ± 4.3	35.1 ± 5.7	0.754
Serum globulin (g/L)	26.3 ± 6.7	27.1 ± 8.1	0.693
BUN (umol/L)	3.76 ± 1.44	3.12 ± 2.09	0.104
pSCr (umol/L)	826 ± 315	795 ± 437	0.430
SCr (umol/L)	551 ± 335	490 ± 445	0.353
UA (umol/L)	417 ± 188	470 ± 135	0.329
Disorders of Ca/P	12 (75.0%)	7 (58.3%)	0.218
CRP (mg/L)	25.7 (2.1 ~ 102.6)	8.8 (0.1 ~ 53.6)	0.102
High CRP	12 (75.0%)	6 (50.0%)	0.432
CD4 (n/ml)	433 ± 182	367 ± 187	0.306
CD8 (n/ml)	386 ± 233	325 ± 397	0.071
Crescent (%)	81.5 (68.1 ~ 100)	60.8 (45.8 ~ 87.9)	0.056
Cellular crescent (%)	17.4 (1.3 ~ 32.3)	14.8 (1.1 ~ 75.8)	0.536
Fibrous/ fibrocellular crescents (%)	52.25 (39.2 ~ 76.6)	41.1 (12.7 ~ 75.8)	0.538
Vascular loop necrosis	11 (68.8%)	9 (75.0%)	0.655
Bowman’s wall fracture	14 (87.5%)	11 (91.7%)	0.691
Acute interstitial lesions	2.00 ± 0.73	1.50 ± 1.24	0.251
Chronic interstitial lesions	1.88 ± 1.02	2.00 ± 0.73	0.107
Tubular atrophy	2.50 ± 0.73	2.08 ± 1.08	0.520
Interstitial cell infiltration	2.00 ± 0.73	1.50 ± 1.24	0.133
Tubulitis	1.88 ± 1.02	2.00 ± 0.73	0.675
Interstitial Fibrosis	2.50 ± 0.73	2.08 ± 1.08	0.730
Stromal vascular lesions	10 (62.5%)	7 (58.3%)	0.754

SCr, serum creatinine; BUN, bloodureanitrogen; UA, uric acid; pSCr, highest serum creatinine in the course.

### Effects of DFPP and IA

#### Removal of serum antibodies and IgG

**Figure 1:**
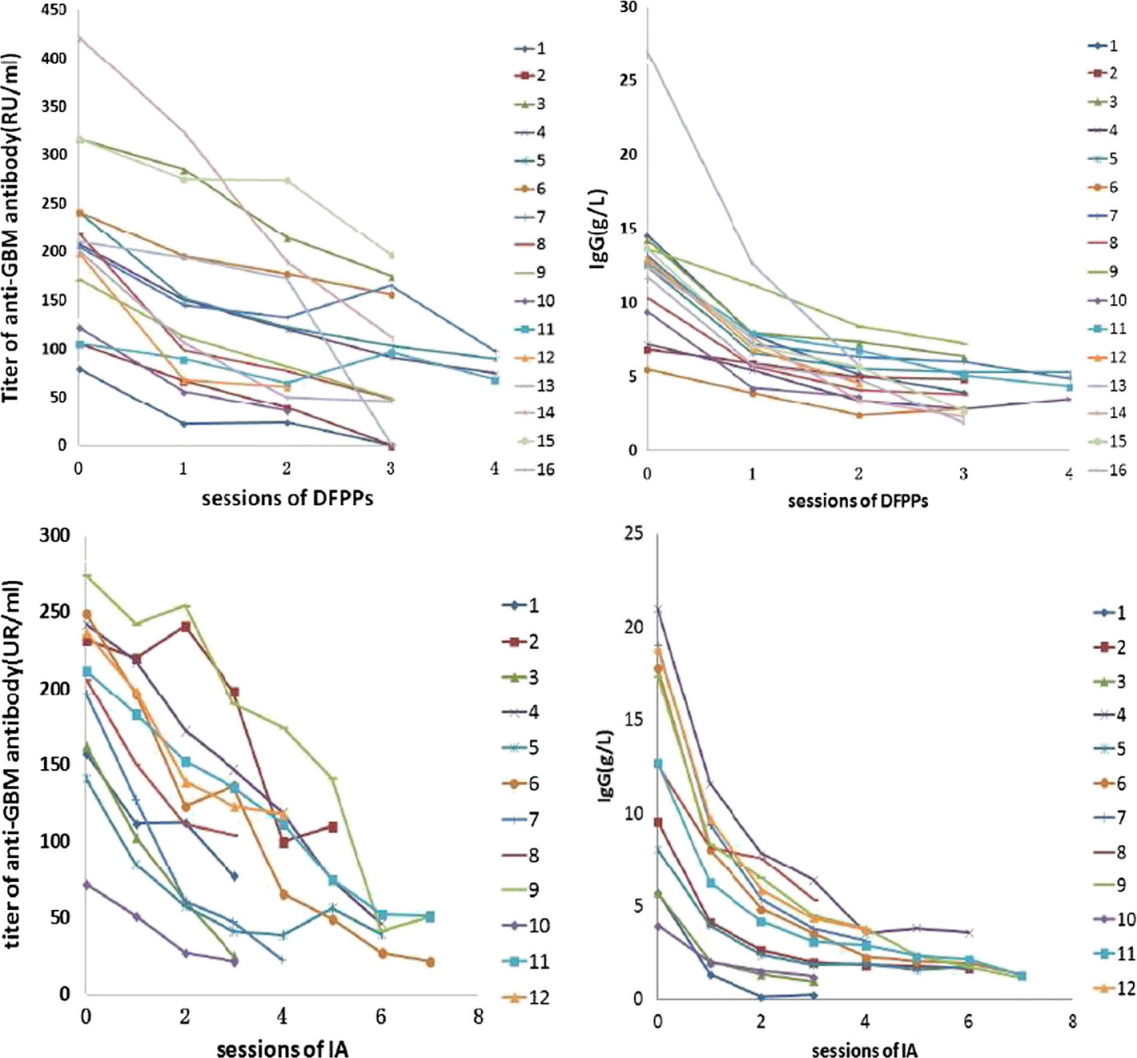
**Effects of IA and DFPP on concentrations of anti-GBM antibodies and IgG in individual patients: In DFPP group, the average reduction of anti-GBM-antibody was 123.7 RU/ml(61.9%), as did the average reduction of IgG was 8.2 g/L(62.7%).** Similarly, in the IA group the average reduction of anti-GBM-antibody was 142 RU/ml(70.8%), as did the average reduction of IgG was 10.6 g/L(82.5%).

Following the DFPP sessions, the concentration of anti-GBM-antibody declined significantly, from 210 to 86.3 RU/ml (P < 0.01), a reduction of 123.7 RU/ml or 61.9%, as did the concentration of IgG, from 12.4 to 4.2 g/L, a reduction of 8.2 g/L or 62.7% (Table 3, Figure 1). Similarly, the concentration of anti-GBM antibody in the IA group declined from 199 to 57.0 RU/ml, a reduction of 142 RU/ml or 70.8%, and the concentration of IgG from 12.7 to 2.1 g/L, a reduction of 10.6 g/L or 82.5%. The change in IgG concentration differed significantly in the two groups (p = 0.049), whereas the change in anti-GBM concentration did not (p = 0.452).

**Table 3:** Comparative effects of IA and DFPP on changes in anti-GBM antibody and IgG concentrations

	**IA**	**DFPP**	**P**
anti-GBM (RU/ml)	141	124	0.452
IgG (g/L)	10.6	8.1	0.049

### Side effects of DFPP and IA

Four patients experienced fever during DFPP, one had bleeding in the lung and one had ecchymosis in the skin. The adverse effect rates were similar in the DFPP and AI groups (Table 4).

**Table 4:** Adverse effects during DFPP and IA

	**DFPP group (n = 16)**	**IA group (n = 12)**	**p**
Fever	4	2	0.701
Bleeding			
Lung	1	1	0.782
Gastrointestinal	0	0	0.302
Skin	1	0	0.622

### Follow-up and outcome

**Figure 2:**
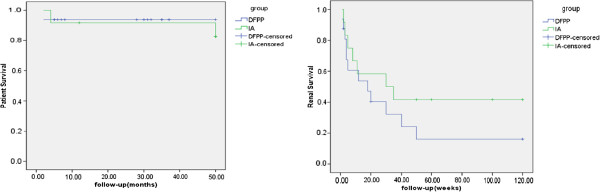
**The patient survive and the renal survive were compared between the DFPP group and the IA group: the patient survival was similar.** The renal survival of IA group was a little better than the DFPP group, but the difference was no significant between the two groups.

Of the 16 patients in the DFPP group, 3 (18.8%) died at presentation or during follow-up; four (25.0%) were lost to follow-up; and nine (59.4%) progressed to end-stage renal disease (ESRD), with a median renal survival of 9 weeks (95% CI, 5–15 weeks). Five patients in this group (31.3%) progressed to CRF, while one returned to normal, being negative for anti-GBM antibody and on urine tests, and an SCr range of 67–86 μmol/L. Outcomes were similar in the two groups (Figure 2).

## Discussion

To our knowledge, this study is the largest to date comparing these two methods of extracorporeal blood purification in patients with anti-GBM disease, including 16 who received DFPP treatment. Although renal dysfunction and abnormal urine analysis did not improve significantly after DFPP, serum anti-GBM-antibody titers declined significantly, demonstrating the effectiveness of DFPP therapy in removing these antibodies. Moreover, the ability of DFFP to remove anti-GBM antibodies was similar to that of IA. The two groups were also quite similar clinically and pathologically, as well as in renal and patient survival outcomes. None of these patients experienced any severe side effects during treatment. DFPP was superior to IA in reducing the loss of useful substances and in requirements for exogenous supplements owing to semi-selective removal. Reductions in IgG concentration were lower with DFPP than with IA, and DFPP was unrestricted, lacking an IA column.

Anti-GBM antibodies initiate the inflammatory destruction of the basement membrane of the kidney glomeruli, leading to a rapidly progressive glomerulonephritis [3]. Treatments are focused on decreasing inflammation induced by circulating antibodies and on removing these antibodies [4]. Although plasma exchange (PE) was effective in removing these antibodies [5], it unselectively removed all plasma proteins. Thus, these patients required replacement of human proteins. Moreover, PE was associated with inherent risks, including anaphylactic reactions, transfusion-related acute lung injury and transfusion-transmitted infections, including infections with hepatitis B and C virus and human immunodeficiency virus. Furthermore, PE required large volumes of plasma and was therefore costly, limiting use of this method [6,7].

DFPP was designed to selectively remove the immunoglobulin fraction from serum, thus minimizing the volume of substitution fluid [8]. Unlike PE, DFPP selectively removes high-molecular-weight molecules, minimizing albumin loss, the need for substitution fluids, and associated hemodynamic fluctuations [9]. DFPP requires only 10–15% of the plasma volume required during PE, greatly reducing the incidence of adverse reactions. Moreover, using albumin or normal saline instead of plasma as a replacement fluid allows DFPP to be performed even in the absence of plasma.

We found that the concentrations of anti-GBM-antibody decreased significantly after DFPP procedures, with a mean decrement of 56.3 ± 22.7%, indicating that DFPP may be effective at clearing anti-GBM antibodies. In comparison, IgG concentration declined a mean 67.4 ± 12.3%. After the first procedure, IgG decreased 37.8 ± 12.4%, whereas anti-GBM-antibody decreased 24.3 ± 16.0%. Thus, in agreement with previous findings, we found that a simple DFPP procedure was more efficient at removing anti-GBM antibodies than IgG [10]. Moreover, in agreement with previous findings [11], we found that serum IgG concentration decreased exponentially while serum anti-GBM antibodies decreased linearly.

None of the patients in the DFPP group experienced severe complications. Four patients had fever during the course of DFPP, but their temperatures decreased to normal range after antibiotic therapy. Nevertheless, it was difficult to determine whether fever was caused by infection or an allergic reaction. One patient had diffuse alveolar hemorrhage, as confirmed by chest CT, but without severe hemoptysis, with the symptoms improving after symptomatic treatment. One study of 335 patients who underwent a total of 2502 plasmapheresis procedures during 515 courses of plasmapheresis found that complications during DFPP included hypotension, bleeding events, allergic reactions, muscle cramps, catheter-related complications such as vascular trauma and septicemia, and problems associated with membrane filters such as hemolysis [12]. That study found that 67.5% of patients experienced complications, occurring during 26.3% of procedures and 60.0% of courses of plasmapheresis.

Hemolysis may be the most frequent complication of DFPP, reported during 20% of procedures. While only 2 of our patients had visible hemolysis that affected the skin or lungs, slight hemolysis without an obvious decrease in hemoglobin (Hb) concentration may have occurred. The mean decrease in Hb following a course of DFPP was 2 g/L. However, since DFPP could also remove fibrinogen, patients undergoing this procedure may have hypofibrinogenemia. Although this condition is not associated with bleeding tendency, clinically overt bleeding has been occasionally reported, especially after serial plasmapheresis. Moreover, most of our patients had anemia and were treated with erythropoietin. Because of the difficulty determining whether DFPP increased internal bleeding, invasive procedures should not be performed during the course of DFPP and high-risk patients should be closely monitored during DFPP treatment. The time required for fibrinogen concentration to return to pretreatment levels after a single DFPP session is 3 to 4 days. Therefore, all of our patients were administered fresh plasma infusions after DFPP to restore fibrinogen and prevent bleeding episodes, perhaps explaining why none of these patients experienced severe hemolysis during the course of DFPP.

Outcomes were similar in the DFPP and IA groups. We found that 4/16 (25%) patients in the DFPP group no longer required maintenance renal replacement therapy at the end of follow-up, a lower percentage than previously reported [13]. Renal injury, however, was more severe in our patients. For example, the maximum mean SCr concentration was 826 μmol/l, and the highest SCr levels in 14 of the 16 patients in this group were higher than the level indicative of uremia. These 14 patients therefore required renal replacement therapy at admission. In addition, although the prognosis of patients with anti-GBM nephritis would be improved by earlier diagnosis and treatment [14-17], the time from disease onset to admission in our patients ranged from 10 days to 13 months. A comparison of clinical features showed that patients who progressed to ESRD tended to have a longer course of disease. Therefore, a longer time from disease onset to admission is predictive of poorer prognosis. Thus, if the diagnosis is highly suspected, it may be appropriate to begin plasmapheresis while awaiting confirmation. The titer of circulating anti-GBM antibody is not the only determinant of the degree of renal injury. Complement components and proteases are involved in the process of renal injury following the binding of anti-GBM antibody to GBM.

Compared with patients in a previous study [18], our patients had more severe lesions histopathologically. Seven of the 16 (43.8%) patients had >85% crescent formation, indicative of a poor outcome [19,20]. A comparison of the pathological features in patients with different outcomes showed that patients who progressed to ESRD tended to have more crescent formation, especially fibrocellular/fibrous crescents. Taken together, all of the factors cited above may affect the prognosis of renal function and may explain why outcomes in our DFPP group were not better than previously reported [21]. Moreover, urine increased in three patients after DFPP therapy.

Correlation analysis showed that outcomes were significantly and negatively corrected with formation of crescents (r = -0.462, P = 0.008) and fibrocellular/fibrous crescents (r = -0.376, P = 0.034), with peak SCr concentration (r = -0.495, P = 0.004), and with the course of kidney disease (r = -0.393, P = 0.015). The predictors of kidney survival in patients with anti-GBM GN were found to include the percentage of glomerular crescents and SCr concentration and need for dialysis at presentation. We found that all of our patients with an initial SCr >500 μmol/L and 85–100% glomerular crescents became chronically dialysis-dependent despite aggressive treatment.

We found that patient survival was good and kidney survival moderate, suggesting that, unless there was evidence of pulmonary hemorrhage, which indicates a very high risk of death, patients with high initial SCr and crescent formation do not require DFPP or IA. While for the patients with a higher percentage of fresh cellular crescents should be treated with DFPP to remove anti-GBM antibodies, thus avoiding further damage. This is particularly applicable to patients with high serum concentrations of anti-GBM antibodies.

## Conclusion

Compared with IA, DFPP could effectively and safely clear anti-GBM antibodies and to some extent improve renal symptoms. DFPP may be a better choice for the treatment of anti-GBM disease, especially if IA columns and plasma are insufficient. DFPP resulted in good patient survival and moderate kidney survival. Except for patients with pulmonary hemorrhage requiring active plasma therapy, complications during DFPP may be decreased by reducing the number of procedures in patients with SCr >500 μmol/L and severe chronic renal histology, especially those with a long course of disease.

## Competing interests

The authors declare that they have no competing interests.

## Authors’ contributions

ZYY participated in the design of the study, performed the statistical analysis and wrote the manuscript. TZ conceived of the study, and participated in its design and coordination and helped to modificated the manuscript. CDM helped to collect the data and check the analysis and first draft. GDH and JDX provided the support of DFPP protocols. LZH approved the final version. All authors read and approved the final manuscript.

## Pre-publication history

The pre-publication history for this paper can be accessed here:

http://www.biomedcentral.com/1471-2369/15/128/prepub
